# Merck’s Index of Fine Chemicals and Drugs, for the Materia Medica and the Arts

**Published:** 1889-05

**Authors:** 


**Merck's Index of Fine Chemicals and Drugs, for the Materia Medica and the Arts,** comprising a summary of whatever chemical products are to-day adjudged as being useful in either medicine or technology, with average values and synonyms affixed. A guide to the physician, apothecary, chemist, and dealer. First American edition. Cloth, 12mo, pp. 168. $1.00. By E. MERCK. Darmstadt, New York, and London. 1889.

This little book is a most complete compendium of Merck's chemicals, to the number of more than 4,000 different articles. The standard trade value is affixed to each drug and, in many instances, the specific gravity, boiling and melting point, formulæ, etc., are mentioned. The physiological and therapeutic effects of the newer remedies are also given. Synonyms are given when necessary, and each officinal drug has its pharmacopeial name. The book is neat, handy, and cheap. The ruled spaces for notes add to the value of the work. The publisher is E. Merck, the well-known manufacturing chemist of Darmstadt, London, and New York.

